# Lysolecithin Improves Lipid Metabolism and Gut Microbiota: An Integrated Transcriptome and Microbiome Analysis in Largemouth Bass (*Micropterus salmoides*) Fed Stearin-Based High-Lipid Diets

**DOI:** 10.3390/metabo16050297

**Published:** 2026-04-27

**Authors:** Yuexing Zhang, Tianyu Feng, Zhiyong Dong, Tianhong Ke, Trond Storebakken, Wanjie Cai, Bo Shi, Liying Huang

**Affiliations:** 1 National Engineering Research Center for Marine Aquaculture, Marine Science and Technology College, Zhejiang Ocean University, Zhoushan 316022, China; yuexing.zhang@zjou.edu.cn (Y.Z.); 2023110101051@zjou.edu.cn (T.F.); 035454@zjou.edu.cn (Z.D.); tianhong.ke@buhlergroup.com (T.K.); caiwanjie@zjou.edu.cn (W.C.); 2 Department of Animal and Aquaculture Science, Faculty of Bioscience, Norwegian University of Life Science, NO-1432 Ås, Norway; trond.storebakken@nmbu.no; 3 Application R&D Centre for Asian and Pacific, Bühler Group, Liyang 213300, China; 4 Zhejiang Marine Fisheries Research Institute, Zhoushan 316021, China

**Keywords:** lysolecithin, emulsifier, multi-omics, lipid metabolic pathway, gut microbiota, *Micropterus salmoides*, high-lipid diet

## Abstract

**Highlights:**

**What are the main findings?**

- Dietary inclusion of lysolecithin enhanced lipid metabolism in largemouth bass.
- Dietary inclusion of lysolecithin modulated gut microbiota, increasing *Cetobacterium.*

**What are the implications of the main findings?**

- Lysolecithin is a promising additive for stearin-based high-lipid aquafeeds.

**Abstract:**

**Background:** Supplementing aquafeeds with emulsifiers can enhance lipid utilization, yet the physiological effects of lysolecithin, derived from enzymatic lecithin conversion, remain under-explored. **Objectives:** This study examined the effects of lysolecithin supplementation on hepatopancreatic transcriptome and gut microbiota in largemouth bass (*Micropterus salmoides*) fed stearin-based high-lipid diets. **Methods:** Two diets were formulated: a control containing 130 g kg^−1^ stearin fish oil (SO), and in the experimental diet (SL), 3.1 g kg^−1^ rapeseed oil was replaced with 3.1 g kg^−1^ lysolecithin oil. Each diet was fed to three replicate groups for 56 days. Hepatopancreas and distal intestine were sampled for transcriptome profiling, and gut microbiota were characterized at 28 and 56 days. **Results:** Lysolecithin supplementation resulted in 424 differentially expressed genes compared with the control (322 up- and 102 downregulated). KEGG enrichment indicated major effects on lipid metabolic processes, notably activation of the PI3K-AKT signaling pathway, enhanced adipocyte lipolysis, and modulation of adipocytokine signaling, suggesting improved insulin sensitivity and lipid mobilization. Histological analysis showed mild distal intestinal inflammation in the SO group. Gut microbiota composition shifted over time; lysolecithin increased the relative abundance of *Cetobacterium* and reduced potential opportunistic taxa compared with the control. **Conclusions:** Overall, dietary inclusion of lysolecithin improved lipid utilization in largemouth bass, likely by enhancing lipid metabolism and promoting beneficial gut microbial profiles. These findings support lysolecithin as a promising feed additive for optimizing high-lipid aquafeeds.

## 1. Introduction

Aquaculture feed ingredients have long been dominated by fishmeal and fish oil. In 2024, global marine fisheries yielded 92.3 million tons, with 89% of the catch used for human consumption [1]. Of the total, 10.2 million tons were processed into fishmeal and fish oil for animal feeds, with around 5% of the fish oil sourced from low-value bycatch, generating stearin or stearin fish oil as a by-product [2]. Stearin is primarily utilized for forming lipid spray beads for fish larvae, proving effective at encapsulating water-soluble nutrients and being well tolerated by fish [3,4,5]. Despite its availability and advantageous nutritional profile, which includes high levels of unsaturated fatty acids compared with some vegetable oils, the use of stearin in aquafeeds remains limited due to its poor solubility resulting from its high saturated fatty acid content [6,7]. Nonetheless, stearin also contributes to improved water stability in feed pellets, which is beneficial when feeding crustaceans such as shrimp and crab [7].

Emulsifiers are amphiphilic compounds that facilitate the mixing of lipids and water, thus enhancing lipid solubility and digestion [8]. Lysolecithin, enzymatically produced from lecithin, exhibits superior oil-in-water emulsifying properties and has been shown to enhance lipid digestion efficiency significantly [9]. While initially used extensively in livestock and poultry diets, lysolecithin now attracts growing interest in aquaculture, with studies indicating similar benefits across multiple fish species [10,11,12,13]. Previous research demonstrated that lysolecithin supplementation can mitigate the reduction in nutrient digestibility induced by high-lipid diets containing stearin [14]. Specifically, supplementing diets with 3.1 g kg^−1^ lysolecithin oil significantly increased both feed intake and growth rate in largemouth bass, suggesting this dosage represents optimal inclusion level [14]. Based on these promising results, this study supplemented diets with 3.1 g kg^−1^ lysolecithin oil for further investigation.

Largemouth bass (*Micropterus salmoides*) is now a major freshwater species in Chinese aquaculture, valued for its adaptability, rapid growth, and short breeding cycle [15]. The shift to artificial feeds has largely replaced the use of small, fresh-frozen fish. While commercial feeds support robust juvenile growth, they often result in less satisfactory outcomes for larger fish (>200 g), primarily due to increased risk of fatty liver disease [15]. Although partial replacement of fishmeal and fish oil is feasible, achieving extensive substitution without impairing growth or health remains a major challenge. It is widely accepted that lysolecithin improves the digestibility and absorption of fats by increasing their dispersion in the intestine, although its precise mechanism remains unclear. We hypothesize that lysolecithin facilitates fat digestion and absorption by modulating the intestinal microbiota or influencing hepatic lipid metabolism pathways. Accordingly, the present study integrates transcriptomic and gut microbiota analyses to deepen our understanding of the effects of lysolecithin supplementation in stearin-based high-lipid diets in largemouth bass.

## 2. Materials and Methods

### 2.1. Experimental Diets

Based on our previous study, the optimal dosage of lysolecithin is 3.1 g per kg of feed for *M. salmoides* [14]. Two isonitrogenous (~500 g kg^−1^) and isoenergetic (~22 MJ kg^−1^) diets were formulated (Table 1). The control diet (SO) included 130 g kg^−1^ stearin fish oil and 3.1 g kg^−1^ rapeseed oil, while the experimental diet (SL) replaced rapeseed oil with 3.1 g kg^−1^ lysolecithin oil (400 g kg^−1^ purity). Diets were processed as previously described [14], and their chemical compositions were analyzed according to standard protocols [16].

### 2.2. Fish, Feeding Trial, and Sampling

Juvenile largemouth bass (*M. salmoides*) were obtained from a commercial hatchery (Benao Agricultural Co., Ltd., Huzhou, China) and acclimated for four weeks on a commercial diet. A total of 210 fish with an initial body weight of 157 ± 14.9 g were randomly distributed into six tanks (35 fish per tank), with three replicate tanks per dietary treatment. Sex distribution was randomized across tanks. The management of the feeding trial followed established procedures [14]. During the 56-day experiment, water temperature (23–25 °C), dissolved oxygen (>6.0 mg/L), pH (7.0–7.5), ammonia nitrogen, and nitrite (<0.1 mg/L) were monitored daily to ensure optimal conditions. At the trial’s conclusion, three fish per tank were randomly selected and anesthetized with an overdose of MS-222. The distal intestine from these fish (nine per treatment) was aseptically dissected. Segments were fixed in 4% paraformaldehyde (Solarbio, Beijing, China) for histological analysis (n = 9 per treatment). Additionally, intestinal digesta from three fish per tank were pooled (n = 3 per treatment) for 16S rRNA gene sequencing. Intestinal microbiota samples from the distal intestine were collected at both 28 and 56 days, with sample groups labeled SO28, SL28, SO56, and SL56 based on diet and sampling day. The distal intestine is the primary colonization site for intestinal microbiota in fish. Hepatopancreas samples from the same fish were preserved in RNA stabilization solution (Vazyme, Najing, China) for transcriptomic analysis.

### 2.3. Distal Intestinal Histopathology

Distal intestine tissues were fixed in 4% paraformaldehyde for 24 h at 4 °C, dehydrated through a graded ethanol series (70%, 80%, 95%, and 100%), cleared in xylene, and embedded in paraffin. Thin slices of tissue with a thickness of 5 μm were sectioned using a microtome, dried at 37 °C overnight, and stained with hematoxylin and eosin (H&E). Pathological sections were imaged using a Leica DM2500 optical microscope (Wetzlar, Germany). From each group, nine intestinal samples were collected for sectioning. The length and width of all intestinal folds in each sample were determined through quantitative measurement and blind assessment.

### 2.4. Transcriptomics

#### 2.4.1. RNA Extraction, Library Construction, and Sequencing

Total RNA was extracted from tissues using the Trizol method (Invitrogen, Carlsbad, CA, USA). cDNA libraries were constructed and validated with an Agilent 2100 bioanalyzer (Agilent Technologies, Santa Clara, CA, USA). Sequencing was performed on an Illumina HiSeq4000 platform by BGI-Wuhan Technology Service Co., Ltd (Wuhan, China). Transcriptome data are available in the NCBI Sequence Read Archive under accession PRJNA1135354 (SRR29816383–SRR29816388).

#### 2.4.2. Data Analysis of Transcriptome

Sequencing data were filtered using SOAPnuke (v1.5.6) to generate clean reads in FASTQ format [18]. Clean reads were mapped to the reference genome and coding gene set using HISAT2 (v2.1.0) and Bowtie2 (v2.3.4.3) [19,20]. Gene expression levels were quantified with RSEM (v1.3.1), and a heatmap was generated with pheatmap (v1.0.8) [21]. Differentially expressed genes (DEGs) were identified using DESeq2 (v1.4.5) with the thresholds |Log2(fold change)| > 1 and q-value ≤ 0.05 [22]. GO and KEGG pathway enrichment analyses of DEGs were conducted using Phyper, based on the hypergeometric test with a significance threshold of q ≤ 0.05. To validate the transcriptome data, quantitative real-time PCR (qRT-PCR) was performed, confirming consistent gene expression patterns between qRT-PCR and RNA-Seq results (Figure S1). Gene expression analysis was performed using the methods described by Shi et al. [23]. Primer sequences are provided in Table S1, and further details can be found in the Supplementary Materials.

### 2.5. Intestinal Microbial Analysis

#### 2.5.1. DNA Extraction, PCR Amplification, and Sequencing

Microbial genomic DNA was extracted from distal intestinal digesta using the E.Z.N.A.^®^ soil DNA kit (Omega Bio-tek, Norcross, GA, USA). The 16S rRNA gene was amplified using primers 338F and 806R with an ABI GeneAmp^®^ 9700 PCR thermocycler (Applied Biosystems, Foster City, CA, USA). PCR products from each sample were pooled, purified with the AxyPrep DNA Gel Extraction Kit (Axygen Scientific, Union City, CA, USA), and quantified using a Quantus™ Fluorometer (Promega, Madison, WI, USA). Libraries were constructed with the NEXTflexTM Rapid DNA-Seq Kit (PerkinElmer, Waltham, MA, USA). Sequencing was conducted on the Illumina MiSeq PE300 or NovaSeq PE250 platforms at Shanghai Meiji Biomedical Technology Co., Ltd (Shanghai, China). The raw reads were submitted to the NCBI SRA under accession PRJNA1135776 (SRR29825954–SRR29825965).

#### 2.5.2. Data Analysis of 16S rRNA

Raw 16S rRNA sequencing reads underwent demultiplexing and quality filtering with FASTP (v0.19.6), and paired-end reads were merged using FLASH (v1.2.11) [24,25]. Operational taxonomic units (OTUs) were clustered at 97% similarity using UPARSE (v11) [26]. Taxonomic classification was completed with the RDP Classifier (v2.13) and a confidence threshold of 0.7 [27]. Intergroup differences were assessed with Bray–Curtis ANOSIM, while Partial Least Squares Discriminant Analysis (PLS-DA) was used for group discrimination and variable selection. Alpha diversity indices (Ace, Chao, Sobs, Shannon, Simpson, and coverage) were calculated with Mothur (v1.30.2), and beta diversity was evaluated using hierarchical clustering in Qiime (v1.9.1). Differential taxa were identified by LEfSe analysis (LDA score >2, *p* ≤ 0.05).

### 2.6. Statistical Analysis

Histological and gene expression data are presented as mean ± SEM (number of replicates as indicated). Normality (assessed by the Shapiro–Wilk test) and homogeneity of variances (assessed by the Levene test) were tested before the implementation of an independent-samples Student’s *t*-test with SPSS Statistics 20. The significance level was set at *p* < 0.05.

## 3. Results

### 3.1. Transcriptome Analysis

#### 3.1.1. Sequencing and Mapping

As shown in Table S2, six cDNA libraries were constructed (SO-1, SO-2, SO-3, SL-1, SL-2, SL-3). After filtering, each library yielded 42.1–43.3 million clean reads, with over 98.3% aligning to the reference genome. Each sample contained 6.31 Gb of clean bases and a clean reads ratio above 95.2%. Q20 and Q30 scores ranged from 98.8% to 99.0% and 95.6% to 96.4%, respectively, indicating high sequencing quality. Most transcripts exceeded 3000 nt in length (Figure S2). Gene Ontology (GO) annotations and KEGG classifications for all unigenes are presented in Figure S3.

#### 3.1.2. Differential Expression Analysis

A total of 424 genes were significantly differentially expressed between the SO and SL groups (SO vs. SL), including 322 upregulated and 102 downregulated DEGs (Figure S4A). Hierarchical clustering revealed that samples within each group clustered together, exhibiting similar gene expression patterns (Figure S4B).

#### 3.1.3. GO and KEGG Classification

**Figure 1:**
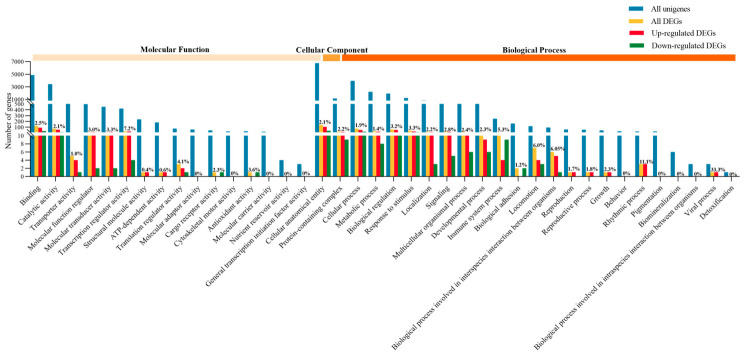
Gene ontology (GO) classification of all unigenes, DEGs, upregulated and downregulated DEGs, and the percentage of DEGs to unigenes (n = 3).

**Figure 2:**
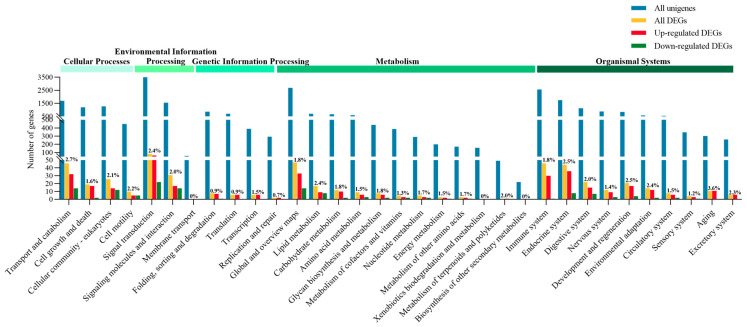
Kyoto Encyclopedia of Genes and Genomes (KEGG) classification of all unigenes, DEGs, upregulated and downregulated DEGs, and the percentage of DEGs to unigenes (n = 3).

All unigenes and DEGs were annotated using the GO database across three main categories: molecular function (16 terms), cellular component (2 terms), and biological process (22 terms) (Figure 1). Most DEGs were assigned to the molecular function and biological process categories, with fewer assigned to cellular components. Within molecular function, the highest ratio of DEGs to unigenes was in transcription regulator activity (7.2%), followed by translation regulator activity (4.1%). In biological processes, viral processes (33.3%) and rhythmic processes (11.1%) had the highest DEG proportions. Similarly, KEGG annotation classified unigenes and DEGs into five categories: cellular processes (4 pathways), environmental information processing (3), genetic information processing (4), metabolism (12), and organismal systems (10) (Figure 2). The majority of DEGs were enriched in metabolism and organismal systems, with fewer in cellular processes, environmental information processing, and genetic information processing. Within metabolism, lipid metabolism had the highest DEG/unigene ratio (2.4%), followed by metabolism of terpenoids and polyketides (2.0%). In organismal systems, the aging pathway (3.6%), the endocrine system (2.5%), and the development and regeneration pathways (2.5%) showed the most notable enrichment. In summary, lysolecithin supplementation resulted in substantial shifts in gene expression, particularly affecting genes related to metabolism, molecular functions, and key biological processes, with a notable emphasis on lipid metabolism and regulatory pathways.

#### 3.1.4. KEGG Pathway Enrichment

**Figure 3:**
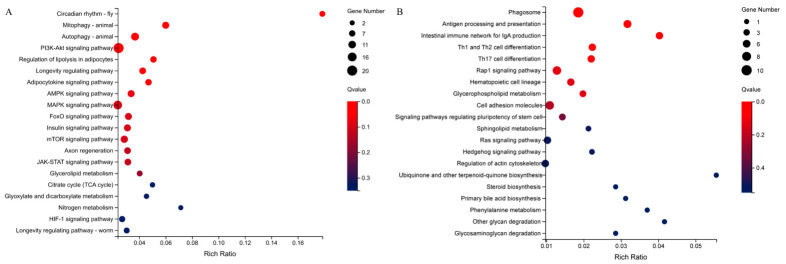
Bubble diagram of KEGG pathway enrichment analysis of upregulated (**A**) and downregulated (**B**) DEGs (n = 3). The x-axis represents the rich ratio, and the y-axis represents the top 20 pathways, respectively. The size and color of the bubble represent the number of DEGs and the q-value, respectively.

Mapping up- and downregulated DEGs to the KEGG database revealed significant pathway changes (Figure 3, Tables S3 and S4). Upregulated DEGs were mainly enriched in seven pathways, including circadian rhythm, mitophagy, autophagy, PI3K-AKT signaling, lipolysis regulation, longevity regulation, and adipocytokine signaling. Downregulated DEGs were primarily associated with phagosomes, antigen processing and presentation, and the intestinal immune network for IgA production (q-value ≤ 0.05).

### 3.2. Histological Characteristics of the Distal Intestine

**Figure 4:**
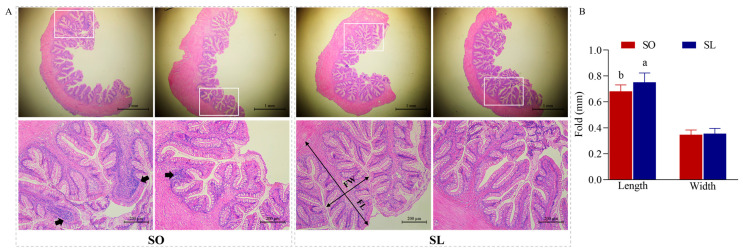
The H & E staining (**A**) and measurement of mucosal folds (**B**) of the distal intestine (n = 9) at 56 days. The black arrows indicate slight infiltration of inflammatory cells. Data are presented as mean and SEM, and different superscript letters indicate significant differences (*p* < 0.05) among treatments. The FL and FW of mucosal folds were analyzed by the TissueFAXS viewer software (Version 7.0). The black arrow shows inflammatory infiltration. FL: fold length, FW: fold width.

Pathological analysis revealed no apparent abnormalities in the SL group at 56 days. At the same time, the SO group showed slight inflammatory cell infiltration in the distal intestine (Figure 4A). Fish fed the SL diet also exhibited significantly greater intestinal fold length compared with those fed SO (Figure 4B, *p* < 0.05).

### 3.3. Gut Microbiota Profile

#### 3.3.1. Similarity Analysis

**Figure 5:**
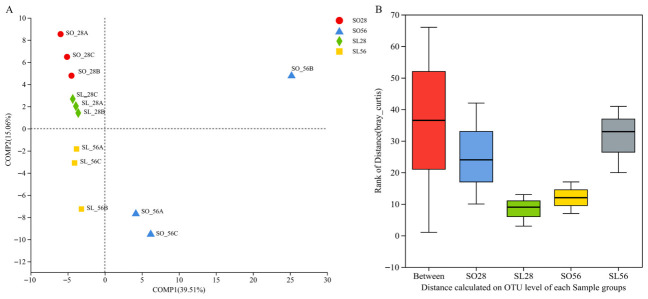
Similarity of bacterial community among samples evaluated by Partial Least Squares Discriminant Analysis (PLS-DA) (**A**) and similarity analysis (ANOSIM, based on Bray–Curtis distance, *p* = 0.038, R = 0.3981) (**B**) (n = 3). Points with different colors represent samples from various groups (left side).

PLS-DA revealed that triplicate samples within each group clustered closely together (Figure 5A). In contrast, ANOSIM analysis indicated that inter-group differences were greater than intra-group variations, as demonstrated by higher Bray–Curtis distances between groups (Figure 5B).

#### 3.3.2. Microbiota Composition

**Figure 6:**
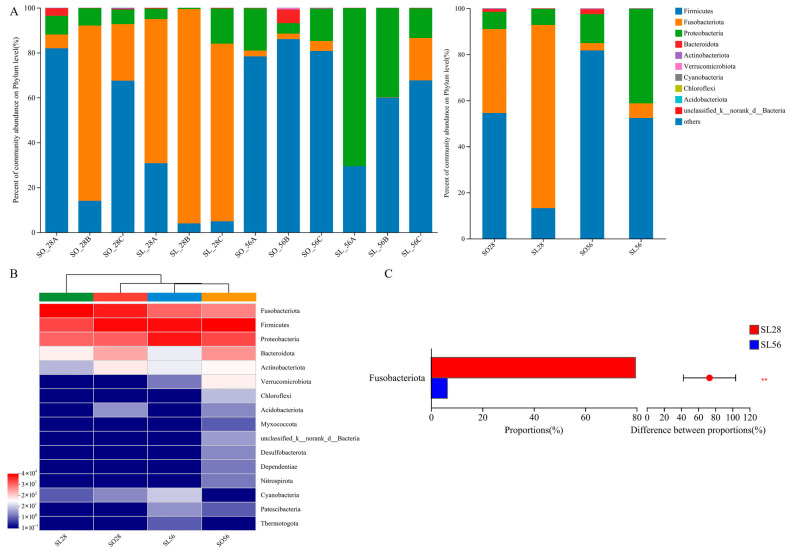
Overview of intestinal microflora abundance at the phylum level (n = 3). (**A**) Abundance of taxa at the phylum level in all samples (left side) and each group (right side); (**B**) Hierarchical cluster analysis with heatmap visualization of taxa at the phylum level in each group; (**C**) Significantly different taxa at the phylum level. The levels of significance were set at *p* < 0.01 (**).

At the phylum level, Firmicutes, Fusobacteriota, and Proteobacteria dominated the gut microbiota, comprising 90% of total abundance (Figure 6A). However, the predominant phyla varied by group: SL28 was dominated by Fusobacteriota, followed by Firmicutes and Proteobacteria. In contrast, SO28, SO56, and SL56 were dominated by Firmicutes, followed by Fusobacteriota and Proteobacteria. Hierarchical clustering revealed the most significant similarity between SO56 and SL56, as well as the most significant difference from SL28 (Figure 6B). The SL28 group exhibited a significantly higher relative abundance of Fusobacteriota compared with SL56 (Figure 6C, *p* < 0.01).

**Figure 7:**
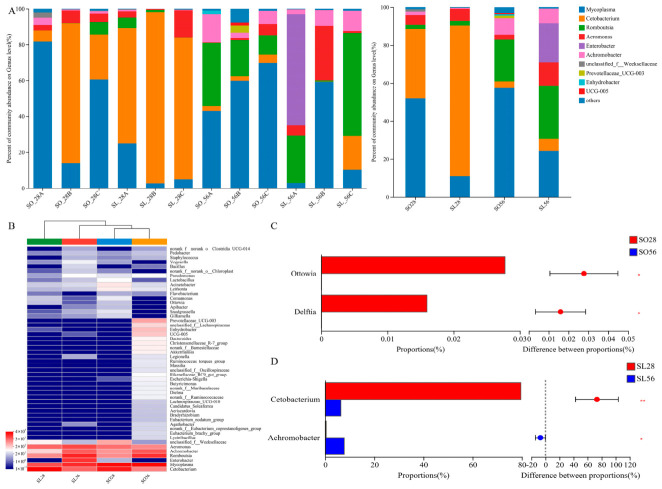
Overview of intestinal microflora abundance at the genus level (n = 3). (**A**) Abundance of taxa at genus level in all samples (left side) and each group (right side); (**B**) Hierarchical cluster analysis with heatmap visualization of taxa at genus level in each group; (**C**,**D**) Significantly different taxa at genus level among different sampling times within the same group. The levels of significance were set at *p* < 0.05 (*); *p* < 0.01 (**).

At the genus level, *Mycoplasma*, *Cetobacterium*, and *Romboutsia* were the most abundant (Figure 7A). In SL28, *Cetobacterium* dominated, followed by *Mycoplasma* and *Aeromonas*. SO28 and SO56 were dominated by *Mycoplasma*, followed by *Cetobacterium* (SO28) or *Romboutsia* (SO56), while SL56 was dominated by *Romboutsia*, then *Mycoplasma* and *Enterobacter*. Genus-level clustering confirmed the highest similarity between SO56 and SL56 and the most significant differences from SL28 (Figure 7B). *Ottowia* and *Delftia* were significantly more abundant in SO28 than in SO56 (Figure 7C, *p* < 0.05). SL28 showed a higher abundance of *Cetobacterium* and a lower *Achromobacter* compared with SL56 (*p* < 0.05).

#### 3.3.3. Alpha Diversity

**Figure 8:**
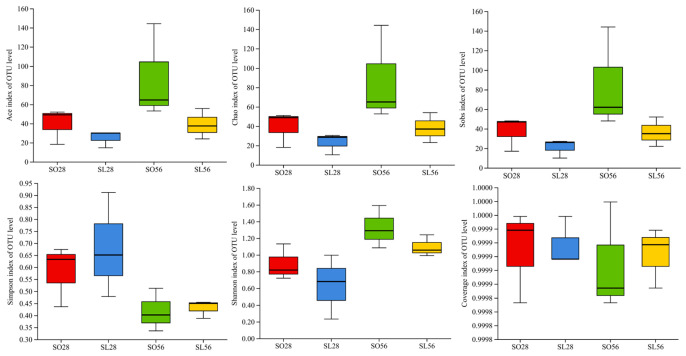
Alpha diversity of intestinal microflora based on Ace, Chao, Sobs, Shannon, Simpson, and Coverage indices (n = 3).

Alpha-diversity indices (ACE, Chao, Sobs, Simpson, and Shannon) showed no significant differences among the groups (Figure 8, *p* > 0.05), indicating that diet and time did not affect overall microbial richness or diversity.

#### 3.3.4. LEfSe Analysis

**Figure 9:**
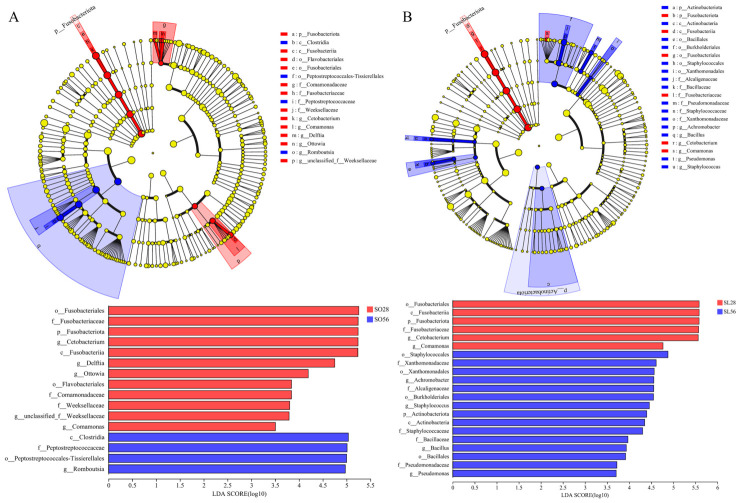
LEfSe analysis of intestinal microflora in SO28 vs. SO56 (**A**) and SL28 vs. SL56 (**B**) (n = 3). LEfSe cladogram, with colored nodes representing microflora taxa significantly enriched in the corresponding groups. Yellow nodes indicate taxa that show no significant difference among the different groups. LDA discriminant bar plot, with higher LDA score indicating greater contribution to group differences (LDA > 2).

**Figure 10:**
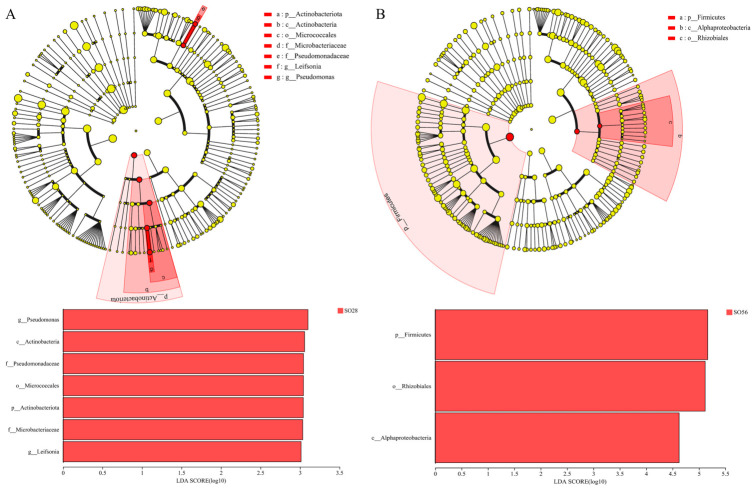
LEfSe analysis of intestinal microflora in SO28 vs. SL28 (**A**) and SO56 vs. SL56 (**B**) (n = 3). LEfSe cladogram, with colored nodes representing microflora taxa significantly enriched in the corresponding groups. Yellow nodes indicate taxa that show no significant difference among the different groups. LDA discriminant bar plot, with higher LDA score indicating greater contribution to group differences (LDA > 2).

LEfSe analysis identified 16 and 21 differentially abundant microbial taxa in SO28 vs. SO56 and SL28 vs. SL56, respectively (Figure 9). SO28 had higher abundances of *Cetobacterium* (*p* = 0.05), *Delftia* (*p* = 0.04), *Ottowia* (*p* = 0.04), and *Comamonas* (*p* = 0.04), while SO56 only had higher *Romboutsia* (*p* = 0.05). SL28 exhibited higher *Cetobacterium* (*p* = 0.05) and *Comamonas* (*p* = 0.05) levels, while SL56 was enriched in *Achromobacter* (*p* = 0.05), *Bacillus* (*p* = 0.04), Staphylococcus (*p* = 0.04), and *Pseudomonas* (*p* = 0.05). At 28 days, the SO diet significantly increased the abundance of *Pseudomonas* and *Leifsonia* (*p* = 0.05, Figure 10).

## 4. Discussion

### 4.1. The Enhancement of Lysolecithin on Hepatopancreatic Insulin Sensitivity and Lipid Utilization Should Be a Key Focus in Future Research

Lysolecithin is a widely used emulsifier in aquafeed that facilitates the formation of hydrophilic micelles, thereby improving nutrient transport across intestinal villi and enhancing the digestion, absorption, and retention of fats and oils [28]. Multiple studies in aquatic species have consistently shown that dietary lysolecithin promotes lipid utilization, growth, and fat absorption. For example, lysolecithin supplementation improved hepatic lipid metabolism in juvenile large yellow croaker [11], enhanced lipid utilization efficiency in turbot [10], increased metabolization and retention of saturated and monounsaturated fatty acids in sturgeon [29], boosted lipase activity in channel catfish [12], and raised fatty acid digestibility in tiger shrimp [28]. The lysolecithin supplementation level in this study was determined based on our preliminary findings, which identified that 3.1 g/kg lysolecithin constitutes the optimal dosage in stearin-based high-lipid diets [14]. In the present study, transcriptomic analysis indicated that the majority of differentially expressed genes (DEGs) in fish fed lysolecithin-supplemented diets were enriched in pathways related to lipid metabolism (KEGG classification). Pathway enrichment specifically identified upregulated DEGs in PI3K-AKT signaling, regulation of lipolysis in adipocytes, and the adipocytokine signaling pathway. The PI3K-AKT pathway is central to diverse physiological processes, including cell survival, proliferation, metabolic regulation, and autophagy [30]. AKT, downstream of PI3K, is critical for insulin signaling and targets regulatory proteins involved in glucose and lipid metabolism [30]. Adipose tissue maintains metabolic balance by regulating triglyceride (TAG) synthesis and breakdown, preventing lipotoxicity, particularly in the liver [31,32]. The adipocytokine pathway involves hormones and cytokines from adipocytes that modulate energy metabolism, inflammation, and immune responses [33]. Further analysis revealed several significantly upregulated DEGs within these pathways, including *foxo3b* (log2(fold change) = 1.5, q = 0.002), *g6pase* (FC = 1.3, q < 0.001), *pck1* (FC = 2.3, q = 0.01), *aqp7* (FC = 2.0, q = 0.04), *pnpla2* (FC = 1.5, q < 0.001), *irs2* (FC = 1.5, q = 0.02), and *adipor2* (FC = 1.8, q < 0.001). The functions of these genes reinforce the effects of lysolecithin supplementation. G6Pase and PCK1, regulated by FOXO, drive hepatic glucose production [34]. AQP7 controls glycerol transport and triglyceride storage in adipocytes [35]. PNPLA2 (ATGL) is the primary enzyme in triglyceride breakdown [36]. IRS-1 and IRS-2 mediate insulin signaling [37], and adiponectin’s insulin-sensitizing role acts through AdipoR1 and AdipoR2 [38]. Overall, the upregulation of these pathways and genes suggested that lysolecithin may facilitate fat digestion and absorption through the enhancement of insulin sensitivity and lipid utilization. These findings provide new directions for subsequent research on lysolecithin.

### 4.2. Lysolecithin Improves Intestinal Morphology and Modifies the Microbiota Profile

Previous studies in broiler chickens have demonstrated beneficial intestinal effects of lysolecithin. Brautigan et al. [39] reported significant increases in villus length and width with commercial and purified lysolecithin supplementation. Similarly, lysolecithin enhanced duodenal cell mitosis [40], and its combination with synthetic emulsifiers and monoglycerides increased jejunal villus height [41]. However, channel catfish fed lysolecithin (125–500 mg/kg) showed no significant changes in intestinal morphology compared with controls [12]. In this study, largemouth bass fed a high-lipid diet without lysolecithin (SO group) exhibited mild inflammatory cell infiltration in the distal intestine. By contrast, fish receiving lysolecithin-supplemented diets showed no intestinal abnormalities and had significantly increased fold length, indicating lysolecithin contributes to the amelioration of high-lipid diet-induced intestinal damage. It should be noted that these histological findings were based on nine intestinal samples per group. Although the quantitative measurement and blind assessment minimized potential bias, a larger sample size would further enhance the reliability of the results. The discrepancy between our findings and those in channel catfish may stem from differences in dietary lipid levels; this study used a high-lipid diet (~17%), while Liu et al. [12] used a normal lipid level (~8%). Previous research suggests lysolecithin is more effective in high-lipid diets [40,42,43,44]. To further explore the effects of lysolecithin, 16S rRNA gene sequencing was used to examine changes in gut microbiota during feeding. The intestinal microbial results presented in this study were derived from three samples per group, a factor that may introduce certain limitations to the data. The microbial composition of the gastrointestinal tract is highly diverse, and the dominant bacterial phyla—Firmicutes, Fusobacteria, and Proteobacteria—were consistent with our prior largemouth bass studies [17,45,46,47,48,49]. Comparative analysis revealed significantly higher *Delftia* abundance, a Proteobacteria member linked to intestinal disorders [50], in the SO group at 28 days versus 56 days. This suggests short-term high-stearin intake (28 days) may promote pathogenic bacteria, which diminish after prolonged feeding (56 days). LEfSe analysis further showed increased *Pseudomonas* abundance in the SO-fed group compared with the lysolecithin group (SL) at 28 days. *Pseudomonas*, a common opportunistic pathogen, can impair intestinal barrier function [51]. The elevated *Pseudomonas* levels correlated with histopathological intestinal damage, reinforcing that lysolecithin supplementation is associated with intestinal health by modulating the microbial environment and reducing the proliferation of pathogenic bacteria.

## 5. Conclusions

This study integrated hepatopancreatic transcriptome and gut microbiota analyses to reveal the effects of lysolecithin supplementation in largemouth bass fed a stearin-based high-lipid diet. KEGG pathway analysis showed that most DEGs were involved in lipid metabolism. Lysolecithin appeared to enhance hepatopancreatic insulin sensitivity and lipid utilization and improve intestinal morphology by increasing fold length in the distal intestine. Short-term high stearin intake promoted the growth of pathogenic *Delftia*, while lysolecithin supplementation reduced the abundance of the conditional pathogen *Pseudomonas*, associated with better intestinal health. The above results are derived mainly from transcriptomic and 16S rRNA gene sequencing data, without additional experimental verification, constituting a limitation of the present study.

## Figures and Tables

**Table 1:** Formulations and chemical compositions of the experimental diets (dry matter).

Ingredients, g kg^−1^	SO	SL
Constant ingredients ^†^	996.9	996.9
Variable ingredients		
Lysolecithin ^‡^	0	3.10
Rapeseed oil	3.10	0
Chemical composition		
Dry matter	928	935
Crude protein	502	500
Total lipid	168	166
Gross energy, MJ kg^−1^	22.3	22.4

^†^ Constant ingredients (g kg^−1^): low-fat anchovy meal 360, soybean meal 150, soy protein concentrate 150, wheat gluten 60, wheat flour 98.4, tapioca 30, stearin, fish oil 130, yttrium 0.5, premix 18. Vitamin and mineral premixes were described in detail by Zhang et al. [17]. ^‡^ Lysolecithin oil (purity 400 g kg^−1^) was provided by Kemin AquaScience (Zhuhai, China).

## Data Availability

Transcriptomic and intestinal microbial data have been deposited in the NCBI database under accession numbers PRJNA1135354 and PRJNA1135776.

## Supplementary Materials

The following supporting information can be downloaded at https://www.mdpi.com/article/10.3390/metabo16050297/s1, Figure S1: Validation of differentially expressed genes by qRT-PCR (n = 3). *g6pase*, glucose-6-phosphatase catalytic subunit; *pck1*, phosphoenolpyruvate carboxykinase cytosolic 1; *pnpla2*, patatin-like phospholipase domain-containing 2; Figure S2: Length distribution of transcripts, the x-axis represents the transcript length interval, and the y-axis represents the number of corresponding transcripts; Figure S3: GO annotations (**A**) and KEGG classification (**B**) of all unigenes in the transcriptome of *Micropterus salmoides*; Figure S4. Summary (**A**) and heatmap visualization (**B**) of DEGs (SO vs. SL): the blue and red columns represent significantly down- and upregulated DEGs, respectively (n = 3); Table S1: Real-time quantitative PCR primer [52]; Table S2: Quality control results of transcriptome data in *Micropterus salmoides*; Table S3: Upregulated DEGs and enriched pathways (|Log2(fold change)| > 1, q-value ≤ 0.05); Table S4: Downregulated DEGs and enriched pathways (|Log2(fold change)| > 1, q-value ≤ 0.05). Gene expression analysis [53].
