# Supplementary material for: Lysolecithin Improves Lipid Metabolism and Gut Microbiota: An Integrated Transcriptome and Microbiome Analysis in Largemouth Bass (Micropterus salmoides) Fed Stearin-Based High-Lipid Diets
Source: Metabolites. 2026 Apr 27;16(5):297. doi: 10.3390/metabo16050297 (PMC13208315; doi:10.3390/metabo16050297)
Supplement: Supplementary file 1 [file metabolites-16-00297-s001.zip › metabolites-4221803-supplementary.pdf]

Table S1

Real-time quantitative PCR primers

Genes	Seq (5’-3’)	Tm (℃)	Genebank ID/References
ef1α	F: TGCTGCTGGTGTTGGTGAGTT<br>R: TTCTGGCTGTAAGGGGGCTC	63	KT827794.1
g6pase	F: GGGAGTCCAGGTGTGTGTCT<br>R: CAGCGAAGGAGGTCAAGAAG	57	[52]
pnpla2	F: AGCATCCAGTTCACTCTCACC<br>R: GGCTTCATCTCCTCTTCATCA	58	XM_038705351.1
pck1	F: TGCTTGACTGGATGTTCAGG<br>R: TTCCTCACCTCATCCACCTC	59	[52]

Table S2

Quality control results of transcriptome data in *Micropterus salmoides*

Groups	Sample	Total raw reads (M)	Total clean reads (M)	Total mapping (%)	Total clean bases (Gb)	Q20 (%)	Q30 (%)	Clean reads ratio (%)
	SO-1	43.7	42.1	98.7	6.31	98.8	95.7	96.3
SO	SO-2	43.7	42.4	98.8	6.36	98.8	95.6	97.0
	SO-3	43.7	42.4	98.8	6.36	98.8	95.7	97.1
	SL-1	43.7	42.2	98.8	6.34	98.8	95.6	96.7
SL	SL-2	43.7	42.3	98.7	6.35	98.9	95.8	96.8
	SL-3	45.4	43.3	98.3	6.49	99.0	96.4	95.2

Table S3

Up-regulated DEGs and enriched pathways (|Log_2(fold change)| > 1, q-value ≤ 0.05)

Pathway name	Rich ratio	*q*-value	DEGs
Circadian rhythm - fly	0.18	< 0.001	nuclear factor, interleukin 3 regulated, member 6 (*nfil3-6*), hepatic leukemia factor-like (*hlf*), D site albumin promoter binding protein b (*dbpb*)
Mitophagy - animal	0.06	< 0.001	hypoxia inducible factor 1 subunit alpha (*hif1al*), activating transcription factor 4b (*atf4b*), microtubule-associated protein 1 light chain 3 beta (*map1lc3b*), BCL2 like 1 (*bcl2l1*), Cbp/p300-interacting transactivator with Glu/Asp-rich carboxy-terminal domain 4a (*cited4a*), forkhead box O3b (*foxo3b*), v-rel avian reticuloendotheliosis viral oncogene homolog A (*rela*), NBR1 autophagy cargo receptor a (*nbr1a*)
Autophagy - animal	0.04	0.02	hypoxia inducible factor 1 subunit alpha (*hif1al*), unc-51 like autophagy activating kinase 2 (*ulk2*), microtubule-associated protein 1 light chain 3 beta (*map1lc3b*), protein kinase cAMP-dependent catalytic alpha genome duplicate a (*prkacaa*), ras-related protein Rab-8A (*rab8a*), insulin receptor substrate 2-like (*irs2*), filamin A interacting protein 1-like (*filip1l*), tumor protein p53-inducible nuclear protein 2 (*tp53inp*), BCL2 like 1 (*bcl2l1*), DEP domain containing MTOR-interacting protein (deptor)
PI3K-Akt signaling pathway	0.02	0.03	myelocytomatosis oncogene homolog (*mych*), activating transcription factor 4b (*atf4b*), phosphoenolpyruvate carboxykinase 1 (*pck1*), MET proto-oncogene, receptor tyrosine kinase (*met*), fibroblast growth factor receptor 4 (*fgfr4*), protein kinase N2 (*pkn2*), eukaryotic translation initiation factor 4E type 3-like (*eif4e*), BCL2 like 1 (*bcl2l1*), forkhead box O3b (*foxo3b*), STKc_SGK domain-containing protein, erythropoietin a (*epoa*), v-rel avian reticuloendotheliosis viral oncogene homolog A (*rela*), growth hormone receptor-like (*ghr*), glucose-6-phosphatase-like (*G6Pase*), glucose-6-phosphatase catalytic subunit 1a tandem duplicate 1 (*g6pc1a.1*),

			macrophage mannose receptor 1-like (*mmr1*), growth hormone receptor a (*ghra*), fibroblast growth factor receptor 2 (*fgfr2*)
Regulation of lipolysis in adipocytes	0.05	0.03	beta-2 adrenergic receptor-like (*beta 2ar*), adrenoceptor beta 2 surface a (*adrb2a*), patatin-like phospholipase domain containing 2 (*pnpla2*), protein kinase cAMP-dependent catalytic alpha genome duplicate a (*prkacaa*), insulin receptor substrate 2-like (*irs2*), aquaporin 7 (*aqp7*)
Longevity-regulating pathway	0.04	0.04	forkhead box O1 a (*foxo1a*), activating transcription factor 4b (*atf4b*), protein kinase cAMP-dependent catalytic alpha genome duplicate a (*prkacaa*), insulin receptor substrate 2-like (*irs2*), eukaryotic translation initiation factor 4E type 3-like (*eif4e*), forkhead box O3b (*foxo3b*), v-rel avian reticuloendotheliosis viral oncogene homolog A (*rela*), adiponectin receptor 2 (*adipor2*)
Adipocytokine signaling pathway	0.05	0.04	phosphoenolpyruvate carboxykinase 1 (*pck1*), insulin receptor substrate 2-like (*irs2*), protein tyrosine phosphatase non-receptor type 11b (*ptpn11b*), v-rel avian reticuloendotheliosis viral oncogene homolog A (*rela*), glucose-6-phosphatase-like (*G6Pase*), glucose-6-phosphatase catalytic subunit 1a tandem duplicate 1 (*g6pc1a.1*), adiponectin receptor 2 (*adipor2*)

Table S4

Down-regulated DEGs and enriched pathways (|Log_2(fold change)| > 1, *q*-value ≤ 0.05)

Pathway name	Rich ratio	*q*-value	DEGs
Phagosome	0.02	0.01	coronin 1A (*coro1a*), mannose receptor C type 1b (*mrc1b*), thrombospondin 1b (*thbs1b*), RLA class II histocompatibility antigen DP alpha-1 chain-like, H-2 class II histocompatibility antigen E-S beta chain-like, type-2 ice-structuring protein-like (*type-2 isp*), RLA class II histocompatibility antigen DP alpha-1 chain-like, mamu class II histocompatibility antigen DR alpha chain-like
Antigen processing and presentation	0.03	0.01	H-2 class II histocompatibility antigen gamma chain-like, RLA class II histocompatibility antigen DP alpha-1 chain-like, H-2 class II histocompatibility antigen E-S beta chain-like, mamu class II histocompatibility antigen DR alpha chain-like
Intestinal immune network for IgA production	0.04	0.01	RLA class II histocompatibility antigen DP alpha-1 chain-like, H-2 class II histocompatibility antigen, E-S beta chain-like, mamu class II histocompatibility antigen DR alpha chain-like

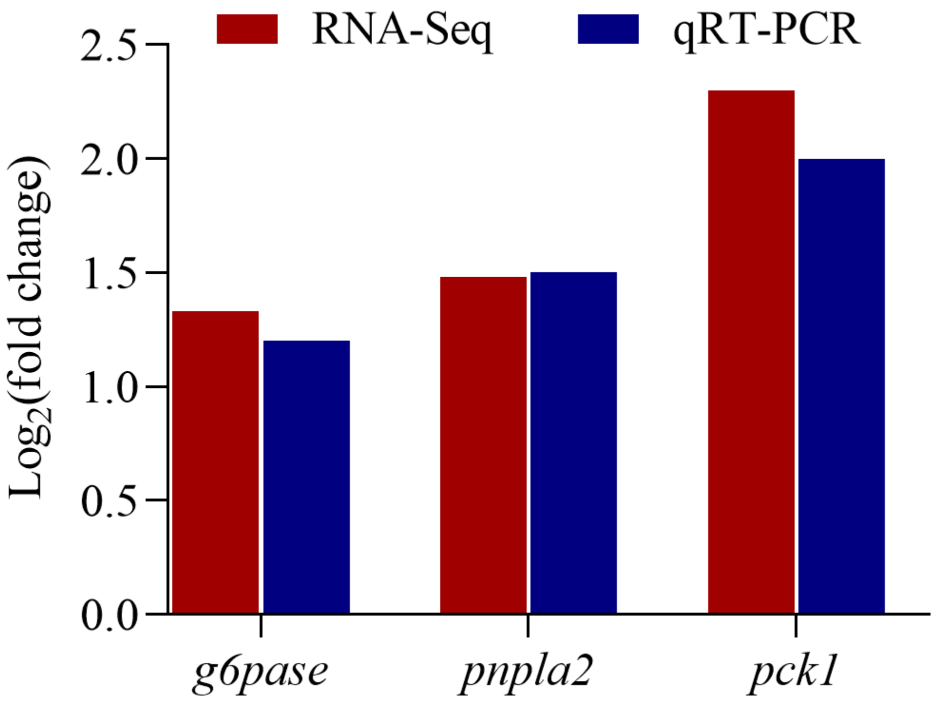

Fig. S1 Validation of differentially expressed genes by qRT-PCR (n=3). *g6pase*, glucose-6-phosphatase catalytic subunit; *pck1*, phosphoenolpyruvate carboxykinase cytosolic 1; *pnpla2*, patatin-like phospholipase domain containing 2.

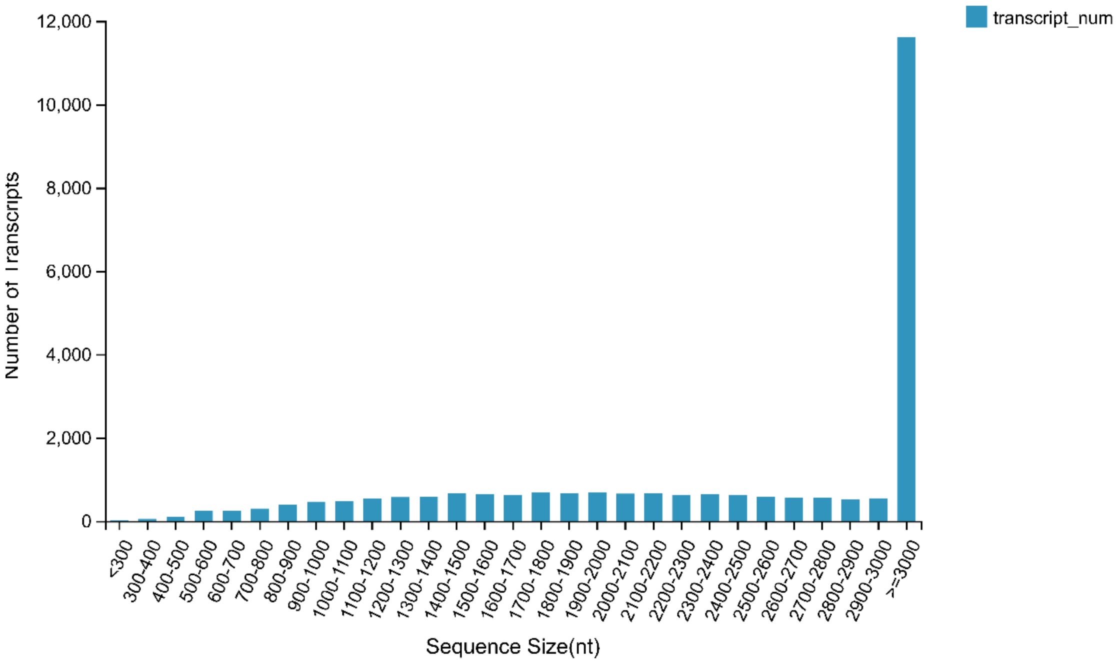

Fig. S2 Length distribution of transcripts, the x-axis represents the transcript length interval, and the y-axis represents the number of corresponding transcripts.

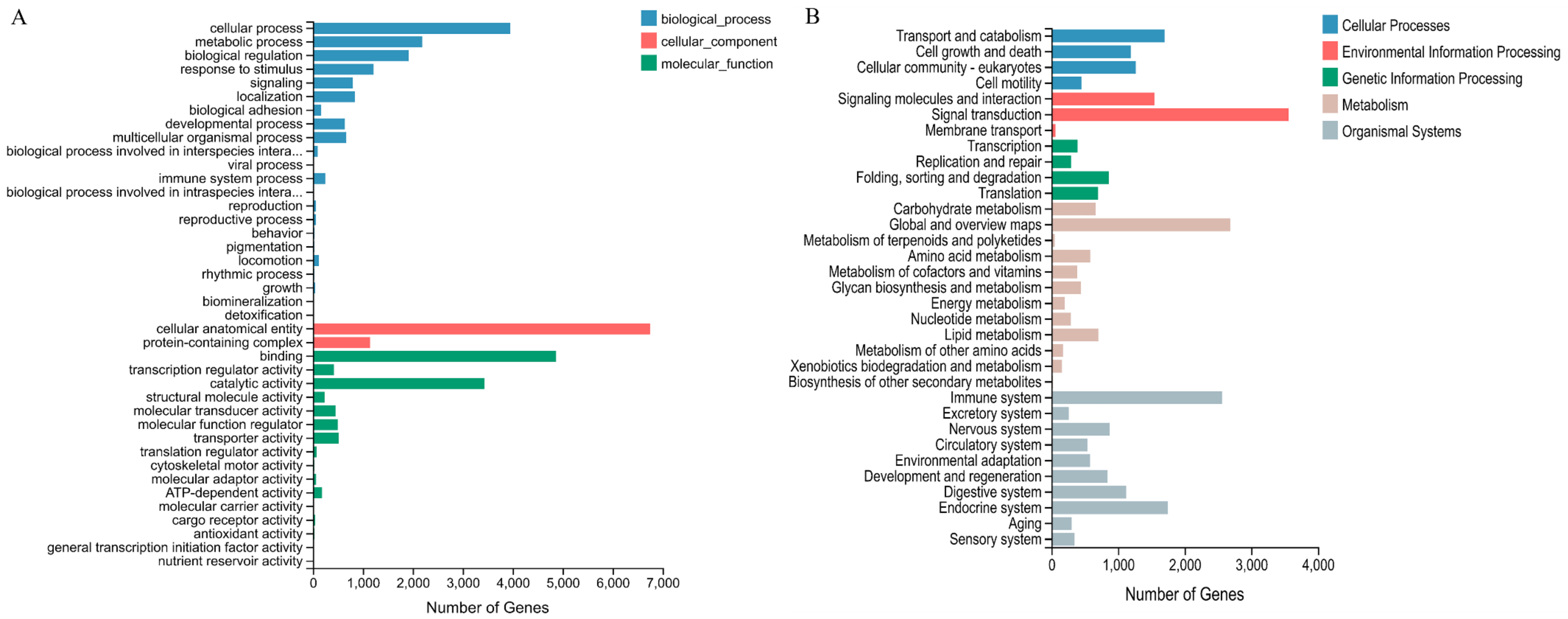

Fig. S3 GO annotations (A) and KEGG classification (B) of all unigenes in the transcriptome of *Micropterus salmoides*

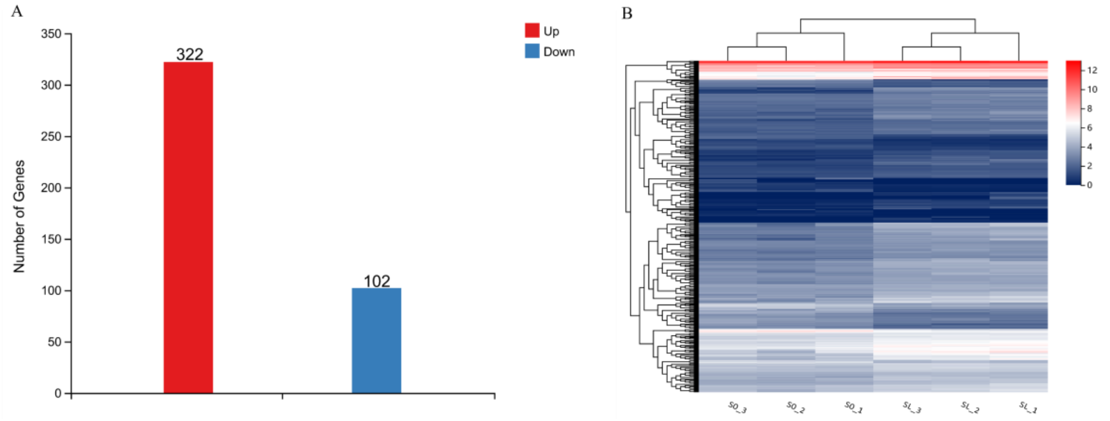

Fig. S4. Summary (A) and heatmap visualization (B) of DEGs (SO vs. SL), the blue and red columns represent significantly down- and up-regulated DEGs, respectively (n = 3).

Gene expression analysis

Total RNA was isolated using a Trizol Reagent (Vazyme, China). The reverse transcription reaction was performed using the HiScript RT SuperMix Reagent kit (Vazyme, China). The real-time quantitative polymerase chain reaction was conducted in a 20 μL reaction system using the ChamQ SYBR qPCR Green Master Mix (Vazyme, China) according to the manufacturer's instructions. A housekeeping gene, *ef1α*, whose expression was found to be unaffected by the treatment in the present study, was used as a reference to normalize the template amount. Data were calculated using the $2^{-\Delta\Delta Ct}$ method [53], with E-values for each gene ranging from 90% to 110%.
